# Effects of a nutraceutical combination on lipids, inflammation and endothelial integrity in patients with subclinical inflammation: a randomized clinical trial

**DOI:** 10.1038/srep23587

**Published:** 2016-03-23

**Authors:** Matteo Pirro, Massimo R. Mannarino, Stefano Ministrini, Francesca Fallarino, Graziana Lupattelli, Vanessa Bianconi, Francesco Bagaglia, Elmo Mannarino

**Affiliations:** 1 Unit of Internal Medicine, Angiology and Arteriosclerosis Diseases, Department of Medicine, University of Perugia, Perugia, Italy; 2 Department of Experimental Medicine, University of Perugia, Perugia, Italy

## Abstract

Cholesterol elevations are associated with systemic inflammation and endothelial fragmentation into microparticles. The cholesterol-lowering efficacy of nutraceutical combinations (NC) has not been investigated in patients with low-grade systemic inflammation and normal-borderline cholesterol levels. This is a 3-month prospective randomized open-label interventional study in patients with elevated plasma high sensitivity C-reactive protein (hsCRP) levels (>2 mg/L) and low-density lipoprotein (LDL) cholesterol of 100–160 mg/dL. The effect of either an oral cholesterol-lowering nutraceutical combination (NC) or no active treatment (noNC) was tested on LDL cholesterol, hsCRP and endothelial microparticle (EMPs) levels. Patients taking the NC had a significant reduction of total (−12%) and LDL cholesterol (−23%) compared to those who received noNC (p < 0.001 for both). Also, hsCRP and EMPs were significantly reduced by the NC (−41% and −16%, respectively). LDL cholesterol change was positively associated with hsCRP (rho = 0.21, p = 0.04) and EMP changes (rho = 0.56, p < 0.001), hsCRP and EMP changes being associated with each other (rho = 0.28, p = 0.005). Patients experiencing both LDL cholesterol and hsCRP reduction were those having the greatest EMP decrease. In conclusion, among patients with low-grade systemic inflammation, an oral NC significantly improved cholesterol profile and attenuated the degree of systemic inflammation and endothelial injury.

Accumulating evidence suggests that atherosclerosis is a chronic inflammatory disease^1^. Accelerated atherosclerosis has been observed in patients with inflammatory diseases, such as rheumatoid arthritis^2^, Sjögren’s syndrome^3^, polymyalgia rheumatica^4^ and psoriasis^5^ and the pro-atherogenic effects of some molecular mediators of inflammation have been postulated^6^. Also, high-sensitivity C-reactive protein (hsCRP) has been used to predict the risk of future cardiovascular events^7^,^8^,^9^.

A relevant pathophysiological mechanism involved in the inflammation-mediated evolution of the atherosclerotic process is represented by the endothelial injury^1^. Endothelial microparticles (EMPs) are small debris derived from endothelial cells membrane fragmentation; their release into circulation occurs in response to either endothelial activation, injury, proliferation or apoptosis^10^,^11^. Elevated EMP levels have been found in acute coronary syndromes^12^, end-stage renal failure^13^, hypertension^14^, hypercholesterolemia^15^ and other conditions^16^. Also, high EMP levels have been found in patients with inflammatory diseases, thus suggesting a causal role of inflammation on EMP formation^4^,^5^,^17^. Importantly, increased plasma EMP levels are associated with a poor cardiovascular prognosis^18^.

Based on current knowledge regarding the detrimental role of inflammation on endothelial integrity^4^,^5^,^17^ and cardiovascular risk^7^,^8^,^9^, targeting systemic low-grade inflammation has become an attractive issue in the prevention of cardiovascular diseases^6^,^19^.

Cholesterol-lowering with statins is a cornerstone of treatment in cardiovascular prevention. The “Air Force/Texas Coronary Atherosclerosis Prevention Study” (AFCAPS/TexCAPS)^20^ and the “Justification for the Use of statins in Prevention: an Intervention Trial Evaluating Rosuvastatin” (JUPITER)^21^, have demonstrated that statin therapy can reduce cholesterol and hsCRP levels, these reductions being associated with a significant improvement of cardiovascular risk. Interestingly, patients with high hsCRP levels obtained a relevant prognostic benefit from statin therapy^20^,^21^. Hence, it is believed that attenuation of inflammation by cholesterol-lowering may have a positive influence on vascular injury^22^ and cardiovascular prognosis^6^.

Despite this evidence, there are several issues limiting an adequate control of combined lipid disorders and the connected low-grade systemic inflammation: these include lack of combination therapies^23^, statin intolerance leading to limited adherence and compliance and consequent drug discontinuation with poor control of plasma lipid levels in specific populations^24^,^25^. With this backgound, novel and safe tools to facilitate the control of lipid abnormalities and of low-grade systemic inflammation are desirable.

Some nutraceuticals have become an attractive tool for cardiovascular prevention because they have been successfully used to treat patients with high cholesterol levels^26^. Red yeast rice, the product of fermentation of rice by the yeast Monascus purpureus, contains monacolins and other substances with cholesterol-lowering efficacy^26^. Particularly, by inhibiting hydroxymethyl-glutaryl-coenzyme-A-reductase (HMGCoAr), monacolin K reduces cholesterol synthesis with a statin-like mechanism of action^27^. Berberine is an herbal extract which is claimed to have significant lipid-lowering properties^26^,^28^. Policosanol is a mixture of long-chain aliphatic alcohols, whose cholesterol-lowering properties are still controversial^26^,^29^. Interestingly, red yeast rice, berberine and policosanol showed anti-inflammatory and/or anti-oxidative effects^30^,^31^,^32^ other than cholesterol-lowering effects^26^,^27^,^28^,^29^. Also, astaxanthin, a lipophilic, pinkish-orange carotenoid, demonstrated both anti-oxidative and anti-inflammatory activity^33^.

The combination of the latter nutraceuticals with folic acid and coenzyme Q10 reduced low-density lipoprotein (LDL) cholesterol in patients with hypercholesterolemia^34^,^35^,^36^,^37^,^38^,^39^ and improved endothelial function and aortic stiffness^34^,^35^. However, there is evidence that cholesterol-lowering is directly related to pre-treatment cholesterol levels^34^,^35^,^36^,^37^,^38^,^39^; also, inflammation reduced the degree of cholesterol-lowering by inducing HMGCoAr resistance^40^. Finally, the ability of this nutraceutical combinations (NC) to reduce hsCRP and EMP levels in patients with low-grade systemic inflammation has not been tested so far. Hence, whether this NC holds its cholesterol-lowering efficacy and reduces systemic inflammation and endothelial fragmentation in patients with elevated hsCRP levels and normal-borderline cholesterol levels need to be clariifed.

We investigated in a 3-month prospective randomized open-label intervention with blinded end-point evaluation the effect of an oral NC containing red yeast rice, berberine, policosanol, astaxanthin, folic acid, coenzyme Q10 on lipid profile, hsCRP and EMP levels in patients with sub-clinical systemic inflammation and normal-borderline cholesterol levels.

## Materials and Methods

The study consisted of a 3-month prospective randomized open-label intervention with blinded end-point evaluation in 100 patients who had been referred to our Lipid Clinic for cardiovascular risk assessment (University of Perugia, Italy), since january 1^st^ 2015 to march 30^th^ 2015. Participants recruitment was planned to be extended after the sample size had been reached until march 30^th^ 2015. Patients (age ranging 25 to 75 years) were enrolled if they had serum hsCRP levels >2 mg/L, LDL cholesterol of 100–160 mg/dL, were not treated with lipid-lowering drugs since at least 6 months before the study starting. Patients with a history of cardiovascular disease or with any coronary risk equivalent (stroke or transient ischemic attack, peripheral arterial disease, aortic abdominal aneurysms, diabetes, multiple risk factors and 10-year Framingham risk >20%) were excluded (risk assessment for determining 10-year cardiovascular risk was carried out according to Framingham risk scoring^41^). Additional exclusion criteria were secondary hyperlipidemia caused by renal, liver or thyroid diseases, alcohol consumption of >40 g/d. None of the participants was receiving drug treatment with anti-platelet, anti-inflammatory, hypolipidemic agents, or hormone replacement therapy.

At the screening visit, a low-cholesterol/low-saturated fat diet, to be followed for 4 weeks before the randomization and to be continued during the 3-month study period, was recommended to all participants. Fifty patients were assigned to 3-month once-daily oral therapy with a patented proprietary NC: red yeast rice extract 200 mg (equivalent to 3 mg monacolins), policosanol 10 mg, berberine 500 mg, astaxanthin 0.5 mg, 0.2 mg folic acid and coenzyme Q10 2 mg (Armolipid Plus^®^, Rottapharm SpA, a MEDA Company). Fifty controls did not receive the NC treatment (noNC). Each patient in the NC arm was supplied with the study NC sufficient for the whole study period. All the participants were instructed to maintain their habitual pattern of physical activity during the duration of the study. Seventeen patients in the NC arm and 14 patients in the noNC arm were on stable anti-hypertensive treatment since at least 6 months.

This study was conducted according to the guidelines laid down in the Declaration of Helsinki and all procedures involving human subjects/patients were approved by the University of Perugia ethical review board (Protocol number 2014–009). Written informed consent was obtained from all subjects/patients. The trial has been registered in ClinicalTrials.gov website (ClinicalTrials.gov Identifier: NCT02422927; URL: https://clinicaltrials.gov/ct2/show/NCT02422927; release date 04.16.15.).

## Clinical Evaluation

All the determinations were made at the medical center at 8:00 AM, with a room temperature between 21 °C and 23 °C, after a 14-hour overnight fast. Height and weight were measured to the nearest 0,1 cm and 0,1 Kg respectively, subjects were wearing hospital gowns and had bare feet. BMI was calculated as weight in kilograms divided by height squared in meters and waist circumference was measured. Brachial blood pressure was measured by a physician with a mercury sphygmomanometer after patients sat for 10 minutes or longer. The average of 3 measurements was considered for the analysis.

### Laboratory parameters

Total cholesterol, triacylglycerols, and high-density lipoprotein (HDL) cholesterol were determined by enzymatic-colorimetric method (Dimension Autoanalyzer; DADE Inc, Newark, NJ); LDL cholesterol was calculated by the Friedewald equation in all participants because none of them had triacylglycerol levels >400 mg/dL. Plasma hsCRP levels were measured using the hsCRP Assay (Siemens Dade Behring, Siemens S.p.A, Milano, Italy) by nephelometry (BN100; Siemens Dade Behring, Siemens S.p.A, Milano, Italy).

Circulating EMPs were detected as previously described^4^,^5^. Platelet-poor plasma was incubated with anti-CD31-PE (Beckman Coulter Inc., Fullerton, CA, USA) plus anti-CD42-FITC (Beckman Coulter Inc., Fullerton, CA, USA.) for 20 min with gentle orbital shaking. Then, phosphate-buffered saline was added, and the sample was ready for flow cytometry on a Coulter Epics XL cytometer (Beckman Coulter Inc.). EMPs were defined as CD31+/CD42− particles with a diameter <1.5 μm (calibration with flow cytometry size calibration beads; Molecular Probes; Invitrogen, Eugene, OR, USA) and the number of CD31+/CD42− microparticles per microliter (microL) of platelet-poor plasma was calculated. Test reproducibility was confirmed by a close correlation (r = 0.91; p < 0.001) between two measurements of CD31+/CD42- EMPs in 20 healthy controls.

### Statistical analysis

SPSS statistical package, release 16.0 (SPSS Inc, Chicago, III) was used for all statistical analyses, with data being analyzed anonymously and by original assigned groups. Values are expressed as the mean ± standard deviation (SD) and the median and interquartile range. Independent-sample t-test and Wilcoxon rank-sum test were used to compare the study variables between patients assigned to either the NC or the noNC arm. Correlation analyses were performed using either Pearson’s or Spearman’s coefficients of correlations. Base 10 logarithmic (log) transformation was performed for skewed variables and the log-values were used. Linear regression analysis was used to predict baseline log-hsCRP levels by including the following independent variables: age, gender, waist circumference and baseline LDL cholesterol levels. Additional regression models with log-EMP levels as dependent variable were carried out by including or not log-hsCRP levels as independent variable; nested model comparisons using the R^2^ change F-test was used to compare models including or not plasma log-hsCRP levels. Paired two-tailed t-tests were performed to assess the influence of either NC or noNC therapies on the study variables. Two-ways analysis of variance for repeated measures (General Linear Model, GLM) was used to compare the response of the study variables to the NC or noNC treatments. Changes of the study variables after the 3-month therapy (Primary outcome measure: LDL cholesterol. Secondary outcome measures: hsCRP and EMPs) was calculated as the difference between baseline values and those at the end of the study intervention. Linear regression analysis was carried out to predict LDL cholesterol variation by including the following independent variables: age, gender, baseline LDL cholesterol levels, log-hsCRP levels and treatment group. An additional regression model was performed for prediction of log-hsCRP variation by the following independent variables: age, gender, baseline log-hsCRP, baseline LDL cholesterol levels and post-treatment LDL cholesterol variation.

We planned a 2-arm, restricted-randomised (random permuted blocks with phone central randomization), parallel group trial of a continuous response variable (LDL cholesterol). Random allocation sequence, participants enrollemnt and treatment assignment were done by different subjects. If the difference in the response of groups would have a standard deviation of 20 and the true difference in the mean response would be 20 mg/dL, assuming a withdrawal/non-evaluable subject rate of 20%, we should need to study 37 pairs of subjects to be able to reject the null hypothesis that this response difference is zero with probability (power) 0.9; the type I error probability associated with this test of this null hypothesis would be 0.01.

## Results

Clinical characteristics of the 100 hypercholesterolemic patients on stable low-fat diet, 50 assigned to oral NC treatment and 50 to noNC, are summarized in Table 1. No patient was either lost or excluded after randomisation. The treatment randomization allowed a balanced distribution of the risk factors between the two study arms.

### Effect of NC on outcome measures

The effect of the 3-month NC or noNC therapy on blood lipids is reported in Table 2. The total and LDL cholesterol concentrations decreased significantly between baseline and 3-month in the NC-treated group compared with the noNC-treated group (p < 0.001 for both GLMs). The reduction of LDL cholesterol was 29 ± 10 mg/dL in the NC-treated group whereas LDL cholesterol did not change (−0.6 ± 13 mg/dL) in the noNC group (Table 2). At 3-month, every patient in the NC group experienced a reduction of LDL cholesterol levels. Triacylglycerol and HDL cholesterol concentrations at 3-month did not differ significantly within or between groups.

At the end of the study intervention, treatment differences of hsCRP and EMPs between NC and noNC were 41% and 16%, respectively (Table 2); GLM for repeated measures was significant for both treatment differences (p = 0.04 and p < 0.001, respectively).

There were no differences in body weight between or within groups at any time.

**Figure 1:**
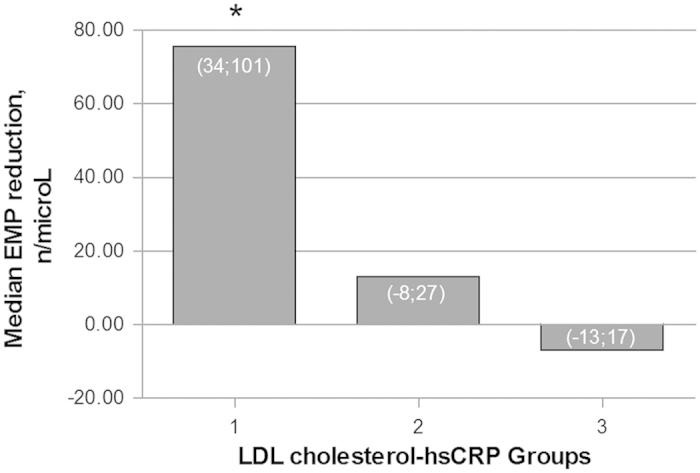
Post-intervention median EMP reduction according to the degree of LDL cholesterol and hsCRP changes. Group 1 includes patients with both LDL cholesterol and hsCRP reductions. Group 2 includes patients with either a LDL cholesterol or hsCRP reduction. Group 3 includes patients without evidence of LDL cholesterol and hsCRP reduction. *p < 0.001 for comparisons between Group 1 and Groups 2 and 3. Values inside the bars indicate the interquartile ranges.

Patients were divided in two groups according to the degree of either the LDL cholesterol or the hsCRP reduction. Group 1 included patients with a LDL cholesterol reduction of at least 15.2 mg/dL (50^th^ percentile of the LDL cholesterol reduction in the entire sample) and a hsCRP reduction ≥ 0.55 mg/L (50^th^ percentile of the hsCRP reduction in the entire sample); Group 2 included patients with either a LDL cholesterol or hsCRP reduction; Group 3 included patients without evidence of LDL cholesterol and hsCRP reduction. Figure 1 shows that patients in the Group 1 had the greatest EMP reduction compared to the other groups.

There were no serious adverse effects in any of the 100 patients randomly assigned. In the noNC-treated group, 2 patients reported minor adverse effects, including headache and palpitations. There were no reported adverse events in the NC-treated group, except for 1 subject who reported an intercurrent and self-limiting gastro-enteritis that was completely resolved in 3 days, during which the patient continued taking the NC. There were no abnormal liver or renal function test nor creatinin kinase elevations at any time for any subject under study.

### Between-variable correlations at baseline and after treatment

At baseline, correlation analyses showed LDL cholesterol was positively associated with hsCRP levels (rho = 0.28, p = 0.005) and EMPs (rho = 0.22, p = 0.02). Also, hsCRP levels were associated with BMI (rho = 0.33, p = 0.001), waist circumference (rho = 0.23, p = 0.02), and EMP levels (rho = 0.31, p = 0.002). Age was a significant covariate of EMP levels (rho = 0.28, p = 0.005).

At the end of the treatment period, LDL cholesterol change was associated with baseline LDL cholesterol (r = 0.20, p = 0.04) but not with baseline hsCRP levels (rho = −0.01, p = 0.95). LDL cholesterol variation was associated with both hsCRP (rho = 0.21, p = 0.04) and EMP changes (rho = 0.56, p < 0.001) and variations of hsCRP and EMP levels were significantly associated with each other (rho = 0.28, p = 0.005).

There was no significant correlation between changes in weight, BMI and waist circumference and either lipids, hsCRP or EMP changes, eliminating body fat change as a possible explanation for the observed changes in the outcome measures.

### Adjusted between-variable associations at baseline and after treatment

At baseline, linear regression analysis with log-hsCRP as dependent variable and age, gender, waist circumference and LDL cholesterol as independent variables showed that both waist circumference (β = 0.24, p = 0.01) and LDL cholesterol levels (β = 0.26, p = 0.007) were associated with log-hsCRP levels (Model R = 0.36, p = 0.001). Linear regression analysis with log-EMP levels as dependent variable was carried out by including as independent variables age, gender, LDL cholesterol (Model 1) and also log-hsCRP (Model 2). Both LDL cholesterol (β = 0.22, p = 0.03) and log-hsCRP (β = 0.22, p = 0.03) were significant predictors of log-EMP levels; nested comparison of the two regression models showed that inclusion of log-hsCRP as an additional independent variable contributed significantly to the improved prediction of the regression model (R^2^ change = 0.045; p = 0.03).

**Figure 2:**
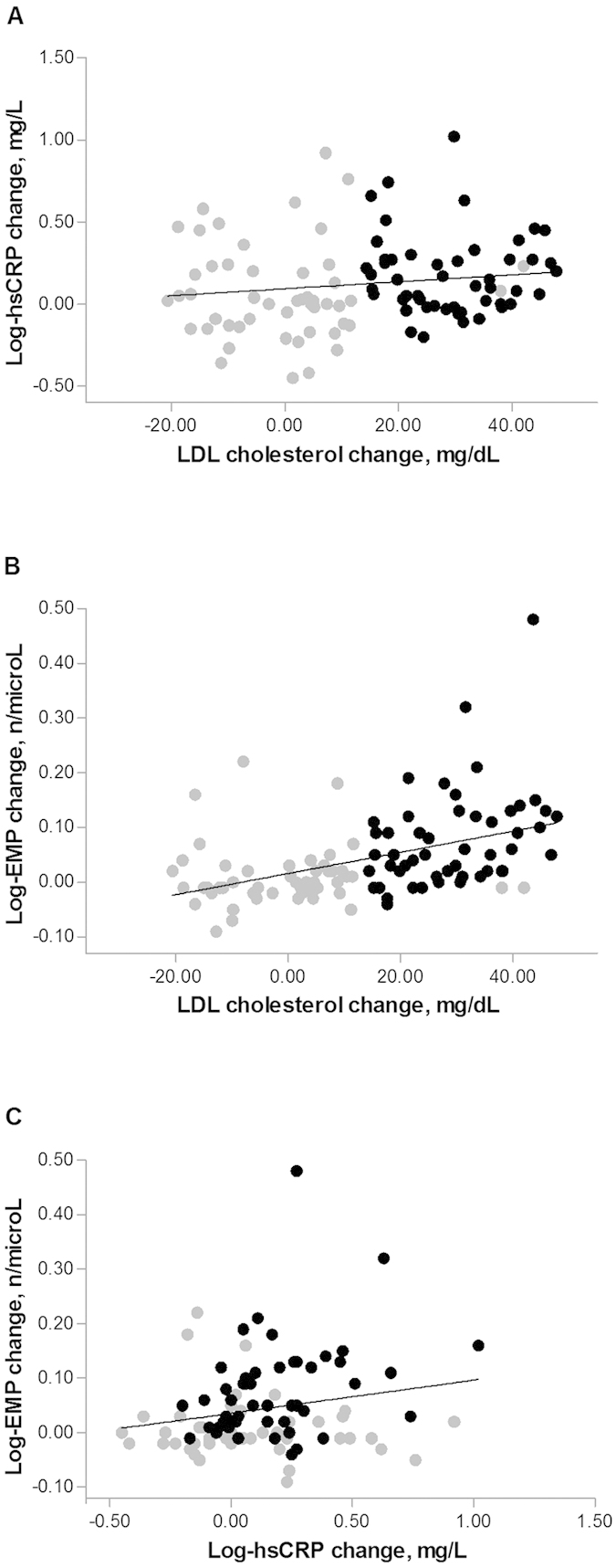
Correlations between LDL cholesterol change and either hsCRP change (**a**) or EMP change (**b**), and between changes in EMP and hsCRP levels (**c**). Data in the entire study population are presented as absolute changes, graphically differentiated according to the treatment group: NC group (black circles), noNC group (grey circles). LDL, low-density lipoprotein; hsCRP, high sensitivity C-reactive protein. EMP, endothelial microparticle.

Correlations of log-variation of hsCRP, EMP and LDL cholesterol in the entire population, differentiated by treatment group, are depicted in Fig. 2.

Linear regression analysis was carried out for prediction of LDL cholesterol change examining the effects of age, gender, baseline LDL cholesterol and log-hsCRP levels; none of these variables were significantly associated with LDL cholesterol variation. When the variable treatment group was also included as an independent variable, it was the only significant predictor of the LDL cholesterol change (β = 0.78, p < 0.001).

Linear regression analysis with log-hsCRP variation as dependent variable and age, gender, baseline log-hsCRP, baseline LDL cholesterol and post-treatment LDL cholesterol variation as independent variables, showed that baseline LDL cholesterol was associated negatively (β = −0.27, p = 0.007) whereas LDL cholesterol change positively with log-hsCRP variation (β = 0.20, p = 0.04). When the variable treatment group was included in the regression model as an independent variable, the significance of the association between LDL cholesterol variation and log-hsCRP variation was lost, while being significant the association between treatment group and log-hsCRP variation (β = 0.22, p = 0.026).

## Discussion

Previous studies in patients with hypercholesterolemia have investigated the ability of an oral NC containing red yeast rice, berberine, policosanol, astaxanthin, folic acid, coenzyme Q10 to reduce cholesterol levels over short periods of treatment^34^,^35^,^36^,^37^,^38^,^39^. Overall, these studies found that this NC, in part through inhibition of HMGCoAr^26^,^42^, was able to reduce total and LDL cholesterol levels^34^,^35^,^36^,^37^,^38^,^39^, with a trend towards greater efficacy in patients with higher pre-treatment LDL cholesterol level^39^. Accordingly, in those studies where pre-treatment LDL cholesterol levels ranged from 150 mg/dL to 175 mg/dL, the NC reduced LDL cholesterol from 15% up to 23%^34^,^35^,^36^,^37^,^38^. The same NC reduced LDL cholesterol by 32% in patients with primary hypercholesterolemia who had a mean baseline LDL cholesterol level above 200 mg/dL^39^, thus suggesting a possible direct association between baseline cholesterol levels and the degree of cholesterol reduction obtained after the NC treatment.

On the basis of these premises, a possible loss of cholesterol-lowering efficacy of an oral NC might be hypotesized in patients with normal-borderline cholesterol levels. Moreover, evidence showing that inflammatory stress mitigated the cholesterol-lowering obtained from HMGCoAr inhibition^40^ might suggest the hypotesis of an additional loss of cholesterol-lowering efficacy of the aforementioned NC.

Despite this background, we found for the first time that a 3-month NC treatment in patients with increased hsCRP levels and normal-borderline cholesterol levels reduced LDL cholesterol by 23% compared to noNC. This result is in line with previous observations by our and other groups in patients with mild cholesterol elevations^34^,^35^,^36^,^37^,^38^. Interestingly, in the present study, the multivariate regression analysis showed that neither baseline LDL cholesterol nor pre-treatment hsCRP levels interfered with the known cholesterol-lowering efficacy of the NC; moreover, subgroup analyses in patients with either baseline hsCRP above 3 mg/L or LDL cholesterol levels below 130 mg/dL showed that the NC still reduced significantly LDL cholesterol (results not shown). Hence, we suggest a stable cholesterol-lowering efficacy of the studied NC in patients with normal-borderline cholesterol levels and hsCRP levels above 2 mg/L.

There are possible explanations of the retained LDL cholesterol-lowering efficacy of the studied NC. First, Chen *et al*.^40^ found that pro-inflammatory cytokines and lipopolysaccharide (LPS) induced statin resistance by disrupting HMGCoAr feedback regulation; in the present study, the presence of a low-grade systemic inflammation instead of a high-grade inflammation induced by LPS^40^ might have been unable to induce a significant HMGCoAr resistance. Second, additional cholesterol-lowering mechanisms other than HMGCoAr inhibition have been proposed for the components of this NC^42^, including proprotein convertase subtilisin/kexin type 9 inhibition by berberine^43^ and increased sterol excretion by red yeast rice^44^. Third, the presence in this NC of compounds with a possible anti-inflammatory action^33^,^45^,^46^,^47^ might have mitigated HMGCoAr resistance, thus promoting an effective cholesterol-lowering. These explanations are all speculative because they were not proved in the present study.

Another finding emerging from the present study is the NC-mediated reduction of hsCRP levels. We found that hsCRP reduction was achieved in 72% of patients randomized to receive the NC, thus supporting a significant anti-inflammatory effect of this NC. Available literature on the effect of this NC on inflammation is limited to one 8-week randomized, double-blind crossover study by Ruscica *et al*.^36^. The Authors found that this NC administred in patients with the metabolic syndrome reduced LDL cholesterol by 21%, but they failed to find any influence of the NC on hsCRP levels^36^. However, higher baseline hsCRP-levels, longer duration of the active treatment, lower prevalence of smokers and lower HDL cholesterol levels in the present study might explain the different results.

We found a statistically significant bivariate association between LDL cholesterol and hsCRP variations; also, the regression analysis including hsCRP variation as dependent variable, showed that the association between LDL cholesterol and hsCRP variations was lost when the analysis was adjusted for treatment group, which instead was strongly associated with hsCRP variation. Overall, these results suggest a role of NC-mediated cholesterol-lowering in the attenuation of the low-grade systemic inflammation. This observation is in line with previous finding of a consistent association between LDL cholesterol and hsCRP reductions obtained with statin therapy^48^. However, our results suggest also that this NC might exert some anti-inflammatory effects which are independent from cholesterol reduction. Accordingly, this NC contains compound like astaxanthin and coenzyme Q10 without significant cholesterol-lowering properties but with some anti-inflammatory and anti-oxidative effects^33^,^47^,^49^.

Beyond the use as an effective tool for cardiovascular risk prediction^7^,^8^,^9^, hsCRP levels have been used to identify patients who will benefit most from cholesterol-lowering with statins, even when LDL cholesterol levels are not in a pathological range of values^6^,^21^. Investigators in the AFCAPS/TexCAPS study reported that subjects with low LDL cholesterol levels and elevated hsCRP levels at baseline benefited markedly from statin treatment, whereas those with low levels of both LDL cholesterol and hsCRP did not^20^. Moreover, a consistent number of trials demonstrated that statins reduced hsCRP levels^48^. Finally, the JUPITER study demonstrated a tremendous clinical benefit from cholesterol- and hsCRP-lowering in patients with normal cholesterol and elevated baseline hsCRP levels^21^.

Hence, in low risk patients with normal-borderline cholesterol levels and increased hsCRP concentration, the use of this NC might be a safe option for reaching a mild but significant cholesterol and hsCRP reduction. It remains to be defined whether these findings translate into a significant clinical benefit and which inflammatory marker better reflects the state of low-grade systemic inflammation and the response to a nutraceutical intervention^50^.

In this study we explored also the degree of mechanical endothelial injury, by measuring circulating EMPs. EMPs are complex submicron membrane-shed vesicles released into the circulation following endothelium cell activation or apoptosis^10^,^11^. Increasing evidence suggests that EMPs play an important role in the pathogenesis of cardiovascular disease either acting as a marker of endothelial injury or exacerbating disease progression^51^.

With respect to EMPs, interesting findings emerged from this study.

First, baseline LDL cholesterol and hsCRP levels contributed significantly to increase EMP levels; accordingly, adding hsCRP as covariate in the linear regression model that included baseline LDL cholesterol levels improved significantly our ability to predict an increased endothelial injury. This result have several implications. First of all, the presence of elevated hsCRP levels in patients with normal-borderline LDL cholesterol might indicate the presence of an endothelial injury, irrespective of other cardiovascular risk factors. This hypothesis is supported by those studies showing that inflammation might promote EMP formation; accordingly, an increased number of circulating EMPs has been observed in different inflammatory conditions, such as rheumatoid arthritis^2^, Sjögren’s syndrome^3^, polymyalgia rheumatica^4^ and psoriasis^5^, and suppression of inflammation is associated with a reduction in circulating EMPs^4^. Furthermore, it has been demonstrated that CRP and the pro-inflammatory cytokines IL-1, IL-6 and IL-8 enhance the release of EMPs from endothelial cells *in vitro*^52^,^53^. On the other hand, it has been proposed that EMPs might play an active role in inducing and maintaining an inflammatory state^54^.

Another implication derived from this result is that even normal-borderline LDL cholesterol levels might contribute to endothelial fragmentation irrespective of systemic low-grade inflammation and additional confounders. Accordingly, we found a significant association between LDL cholesterol in the range of 100–160 mg/dL and EMPs. Thus, attention should be paid also to LDL cholesterol levels that we do not consider frankly pathological, expecially when these levels are associated with hsCRP elevations.

Second, patients experiencing both significant LDL cholesterol and hsCRP reductions after the NC treatment were those obtaining the greatest reduction in circulating EMP levels.

Hence, we migh speculate that targeting both normal-borderline LDL cholesterol levels and low-grade systemic inflammation might have beneficial effects on the endothelial integrity. In this regard, it has already been observed that lipid lowering drugs, such as statins, can reduce LDL cholesterol, hsCRP and the number of circulating EMPs^21^,^48^,^55^. Hence, to have available a new tool, safe and effective, to control the set of the mentioned risk variables (i.e. cholesterol, hsCRP and EMPs), can represent a valid aid to the management of patients at risk of cardiovascular disease.

Our study has limitations. First, the open-label design is a possible source of bias. Second, mechanistic explanations of the retained LDL cholesterol-lowering efficacy of the studied NC in the presence of low-grade systemic inflammation have not been provided. Also, the use of a combined therapy with multiple compounds does not allow to draw conclusions on whether the beneficial effects on LDL cholesterol levels, hsCRP and circulating EMPs might be ascribed to some of the nutraceuticals or to their combination. The use of single agent preparations should be tested to evaluate their respective efficacy on lowering cholesterol levels and reducing hsCRP and circulating EMPs. Measurement of EMP levels might be improved by additional methods to identify an early endothelial dysfunction^56^. Finally, the suggested anti-inflammatory effect of the NC need to be corroborated by additional *in vitro* and *in vivo* tests exploring deeply the systemic inflammation cascade.

Our results may have a potential clinical relevance. Patients with normal-borderline elevations of plasma cholesterol and with evidence of low-grade systemic inflammation are numerous and at increased cardiovascular risk^20^. The studied NC might represent a novel and safe tool to improve lipid and low-grade inflammation control in those patients in which statin treatment is either not recommended or is not tolerated.

In conclusion, in patients with increased hsCRP and normal-borderline cholesterol levels, treatment with a low-dose nutraceutical combination retains a significant cholesterol-lowering activity and reduces low-grade systemic inflammation. Treatment with the nutraceutical combination was paralleled by a significant improvement of endothelial injury that is dependent on both cholesterol and hsCRP reductions.

## Additional Information

**How to cite this article**: Pirro, M. *et al*. Effects of a nutraceutical combination on lipids, inflammation and endothelial integrity in patients with subclinical inflammation: a randomized clinical trial. *Sci. Rep.* **6**, 23587; doi: 10.1038/srep23587 (2016).

## Figures and Tables

**Table 1:** Characteristics of 100 patients with hsCRP above 2 mg/L randomized to either NC therapy or noNC therapy.

	noNC (N = 50) Mean or median and SD or IQR		NC (N = 50) Mean or median and SD or IQR		p
Age, years	64	5	65	3	0.28
Gender, % women	70	–	70	–	1.00
Smoking, %	16	–	14	–	1.00
Body mass index, kg/m^2^	26.7	3.9	26.5	3.4	0.76
Waist circumference, cm	88	11	90	11	0.23
Systolic blood pressure, mmHg	137	12	138	12	0.68
Diastolic blood pressure, mmHg	81	6	82	8	0.25
Total cholesterol, mg/dL	210	24	211	17	0.79
LDL cholesterol, mg/dL	131	16	134	14	0.43
HDL cholesterol, mg/dL	51	12	51	15	0.97
Triglycerides, mg/dL	110	72–186	115	83–177	0.86
Glucose, mg/dL	90	13	90	11	0.43
Framingham risk score, %	8.0	5.7	8.3	6.2	0.79
hsCRP, mg/L	2.7	2.2–4.9	3.0	2.2–4.2	0.63
EMPs, n/microL	401	298–514	416	302–500	0.82

Values are mean ± standard deviation (SD) except for triglycerides, hsCRP and EMPs expressed as median and interquartile range (IQR). NC, nutraceutical combined therapy; LDL, low-density lipoprotein; HDL, high-density lipoprotein; hsCRP, high sensitivity C-reactive protein; EMPs, endothelial microparticles.

**Table 2:** Influence of either the NC or the noNC therapy on selected variables.

	noNC						NC				
	Before Mean or median and SD or IQR		After Mean or median and SD or IQR		% change	p^&^	Before Mean or median and SD or IQR		After Mean or median and SD or IQR		% change
Total cholesterol, mg/dL	210	24	210	25	0.00	< 0.001	211	17	185	17	−12.32*
LDL cholesterol, mg/dL	131	16	132	18	0.76	< 0.001	134	14	105	15	−21.64*
HDL cholesterol, mg/dL	51	12	52	11	1.96	0.12	51	15	54	13	5.88
Triglycerides, mg/dL	110	72–186	103	78–154	−6.36	0.84	115	83–177	109	76–175	−5.21
Body mass index, kg/m^2^	26.7	3.9	26.6	3.8	−0.37	0.99	26.5	3.4	26.3	3.6	−0.75
Waist circumference, cm	88	11	89	9	1.13	0.83	90	11	91	12	1.11
hsCRP, mg/L	2.7	2.2−4.9	3.4	1.8−5.1	25.92	0.04	3.0	2.2−4.2	2.5	1.3–3.4	−16.67*
EMPs, n/microL	401	298–514	407	278–504	1.50	< 0.001	416	302–500	353	247–438	−15.14*

Values are mean ± standard deviation (SD) except for triglycerides, hsCRP and EMPs expressed as median and interquartile range (IQR). NC, nutraceutical combined therapy; LDL, low-density lipoprotein; HDL, high-density lipoprotein; hsCRP, high sensitivity C-reactive protein; EMPs, endothelial microparticles. *p < 0.05 for comparison between values at baseline and those after the NC treatment. ^&^The p value is for the GLM comparison of variable variations after either NC or noNC treatment.
